# Association between serum total cholesterol levels and the risk of incident heart failure in patients with atherosclerotic cardiovascular disease

**DOI:** 10.3389/fmed.2026.1884333

**Published:** 2026-08-28

**Authors:** Xiaoxiao Wang, Shouling Wu, Haibo Gao, Shuohua Chen, Yang Yang, Qi Zhang

**Affiliations:** 1 The Third Hospital of Hebei Medical University, Shijiazhuang, China; 2 Department of Cardiology, The Affiliated Hospital of Chengde Medical College, Chengde, China; 3 Department of Cardiology, Kailuan General Hospital, Tangshan, China; 4 Department of Cardiology, The Affiliated Hospital of North China University of Science and Technology, Tangshan, China; 5 Department of Cardiology, Tangshan Gongren Hospital, Tangshan, China

**Keywords:** atherosclerotic cardiovascular disease, competing risk model, cox proportional hazards model, heart failure, restricted cubic spline, total cholesterol

## Abstract

**Objective:**

To investigate the association between baseline total cholesterol (TC) levels and incident heart failure (HF) risk in patients with atherosclerotic cardiovascular disease (ASCVD).

**Methods:**

This retrospective cohort analysis was based on the Kailuan Study, a prospective cohort initiated in Tangshan, China. A total of 11,479 patients with first-onset ASCVD between 2006 and 2022 and available post-ASCVD TC measurements were included. Participants were classified into five TC groups: C1 (<3.100 mmol/L), C2 (3.100–<4.100 mmol/L), C3 (4.100–<5.200 mmol/L), C4 (5.200–<6.200 mmol/L), and C5 (≥6.200 mmol/L), with C3 as the reference. Cox proportional hazards models were used to assess HF risk. Restricted cubic spline analyses and Fine–Gray subdistribution hazard models were further performed to evaluate the nonlinear association and account for the competing risk of death.

**Results:**

During 71,406.7 person-years of follow-up, corresponding to a mean follow-up duration of 6.22 years, 610 incident HF cases occurred, with an overall incidence proportion of 5.31% and an incidence density of 8.54 per 1,000 person-years. Incidence densities from C1 to C5 were 14.35, 8.46, 7.32, 7.89, and 12.34 per 1,000 person-years, respectively. In the most comprehensively adjusted model (Model 3), which accounted for demographic characteristics, comorbidities, smoking status, body mass index, laboratory indices, medication use, and follow-up TC-related variables, C1 and C5 were associated with higher HF risk than C3, with *HRs* of 1.578 (*95% CI* 1.160–2.148; *p* = 0.004) and 1.509 (*95% CI* 1.150–1.979; *p* = 0.003), respectively. Restricted cubic spline analysis showed a significant nonlinear, approximately U-shaped association, and competing-risk analyses yielded similar findings.

**Conclusion:**

Baseline TC levels showed a nonlinear association with incident HF risk in patients with ASCVD. High TC may reflect persistent atherogenic risk, whereas the association between low TC and HF may partly reflect residual confounding or reverse causation related to underlying comorbidities, inflammation, nutritional depletion, frailty, or lipid-lowering treatment history. Therefore, low TC should be interpreted as a potential risk marker rather than as a causal factor for HF.

## 1 Introduction

Heart failure is an important clinical syndrome that represents the advanced or terminal stage of various cardiovascular diseases and is one of the major causes of hospitalization, mortality, and increased healthcare burden (1, 2). With population aging, improved survival among patients with cardiovascular disease, and the increasing burden of metabolic disorders, the disease burden of heart failure continues to rise (3). Therefore, identifying individuals at high risk of heart failure and implementing early management of modifiable risk factors are of great importance for improving the long-term prognosis of patients with cardiovascular disease.

Atherosclerotic cardiovascular disease (ASCVD), which refers to a cluster of vascular diseases that mainly consist of coronary heart disease, ischemic stroke, and peripheral arterial disease, is one of the main comorbidities that contribute to the onset of heart failure (4). Patients who suffer from ASCVD usually have a variety of additional risk factors, such as hypertension, diabetes mellitus, dyslipidemia, obesity, chronic inflammation, and renal dysfunction. Such factors can cause heart failure through the acceleration of coronary atherosclerosis, myocardial ischemia, remodeling after myocardial infarction, and myocardial fibrosis (5). Recent studies have shown that patients with established ASCVD have a higher risk of developing HF than individuals without ASCVD (6).

Dyslipidemia is an important risk factor for the development and progression of ASCVD, and serum total cholesterol (TC) is one of the routinely measured lipid parameters in clinical practice (7). Although LDL-C and non-HDL-C are established lipid targets in ASCVD management, apolipoprotein B (ApoB) and remnant cholesterol are also increasingly used to characterize residual atherogenic burden. Remnant cholesterol refers to the cholesterol content of triglyceride-rich lipoproteins and their remnants and is commonly estimated as TC minus LDL-C minus HDL-C (8). Nevertheless, TC remains a widely available, routinely measured, and clinically interpretable lipid parameter in large-scale epidemiological cohorts. In addition, TC reflects the overall cholesterol burden from multiple lipoprotein fractions and may capture both atherogenic lipid burden and poor-health-related low cholesterol status. Therefore, TC was selected as the primary exposure in this analysis, while LDL-C and non-HDL-C were further examined in sensitivity analyses. Higher TC levels usually indicate abnormal cholesterol metabolism and may be accompanied by elevated low-density lipoprotein cholesterol, remnant cholesterol, and other atherogenic lipoproteins, thereby promoting vascular endothelial injury, lipid deposition, inflammatory responses, and plaque instability (9, 10). Therefore, lipid management is an important component of secondary prevention in patients with ASCVD, and appropriate control of cholesterol levels is essential for reducing the risk of adverse cardiovascular events (11).

In any case, the correlation between lipid parameters and heart failure can be more complicated compared to its correlation with ASCVD development. Increased TC may contribute to heart failure by worsening coronary atherosclerosis, triggering myocardial ischemia, and causing ventricular remodeling. Low TC levels do not automatically indicate a decreased heart failure risk either. The presence of low TC in patients with cardiovascular diseases or wasting syndromes can signal undernutrition, systemic inflammation, anemia, frailty, or a higher burden of comorbidity (12). Existing literature shows that TC, low-density lipoprotein cholesterol, and non-high-density lipoprotein cholesterol may affect the heart failure risk independently of each other, and their influence is determined by the patient’s initial metabolic state and disease progression stage (13). In addition, the phenomenon known as the “cholesterol paradox” suggests that, in some patients with heart failure or high-risk cardiovascular conditions, lower cholesterol levels may instead be associated with adverse prognosis (14).

To our knowledge, evidence directly linking TC levels measured after ASCVD onset to the subsequent risk of incident HF remains sparse. Contemporary lipid studies in secondary-prevention populations have primarily focused on recurrent ASCVD events and residual cardiovascular risk associated with inadequately controlled LDL-C, whereas large population-based analyses have mainly evaluated associations of lipid-related risk factors with incident cardiovascular disease and mortality. Moreover, even among cohorts of patients with established cardiovascular disease, recent studies of incident HF have more often examined non-lipid predictors, such as systemic inflammation, rather than TC itself (15–17). Consequently, relatively little is known about whether TC levels measured after ASCVD onset are associated with the subsequent risk of HF. Moreover, TC levels in patients with ASCVD may be influenced by underlying disease severity, nutritional status, systemic inflammation, metabolic abnormalities, and lipid-lowering therapy. Therefore, the association between TC and HF risk may be nonlinear and may also be susceptible to residual confounding or reverse causation.

Accordingly, this current research relied on the data obtained from a former cohort and involved patients with a first occurrence of ASCVD between 2006 and 2022. The serum TC concentration post-ASCVD onset was considered the exposure factor. In order to examine the correlation between serum TC concentrations and the occurrence of incident heart failure, Cox proportional hazards regression analysis and nonlinear dose–response correlation assessment using restricted cubic splines were conducted. Moreover, competing risk models were further carried out to address the possible effect of death on the results. This current research sought to further explore the correlation between serum TC concentrations and the risk of developing heart failure among individuals with ASCVD, offering guidance for heart failure risk screening and personalized intervention.

## 2 Materials and methods

### 2.1 Study design and data source

This retrospective cohort analysis was based on the Kailuan Study, a prospective cohort study initiated in 2006–2007 in Tangshan, China, with biennial follow-up examinations. In addition, the cohort was annually cross-linked to medical records from 11 hospitals, including Kailuan General Hospital and its 10 affiliated hospitals, to ascertain new-onset atherosclerotic cardiovascular disease (ASCVD), heart failure, death, and other follow-up outcomes. Participants who experienced a first-onset ASCVD event between 2006 and 2022 and had available serum total cholesterol (TC) measurements after ASCVD onset were included in the present analysis. Post-ASCVD TC level was considered the main exposure variable, and participants were followed for incident heart failure (HF). The aim of this study was to evaluate the association between different TC levels after ASCVD onset and the risk of incident HF among patients with ASCVD.

This study used existing cohort data, including health examination records, clinical diagnosis and treatment data, laboratory test results, medication information, and follow-up outcome data. No additional intervention was performed during the study. Relevant variables were extracted mainly from previous records and follow-up data. Data collection and follow-up were conducted according to standardized procedures for all participants. The data were organized, checked, and subjected to logical verification before statistical analysis.

### 2.2 Study population

Participants from the Kailuan Study who experienced a first-onset ASCVD event between 2006 and 2022 and had available serum TC measurements after ASCVD onset were eligible for inclusion. ASCVD was defined as myocardial infarction, cerebral infarction, or coronary revascularization after non-acute myocardial infarction. The date of the first available serum TC measurement after the index ASCVD event was defined as the baseline date. Only participants who were free of heart failure before and on the baseline TC measurement date were eligible for inclusion. Participants who developed heart failure before or on the date of TC measurement were excluded to ensure that all heart failure events occurred after exposure assessment. Participants were followed from the baseline TC measurement date until incident heart failure, death, loss to follow-up, or the end of the study period, whichever occurred first. The study endpoint was December 30, 2022.

The inclusion criteria were as follows: (1) first occurrence of ASCVD between 2006 and 2022; (2) available serum TC measurement after ASCVD onset; (3) clearly defined follow-up start and end times and available information on heart failure outcomes; and (4) relatively complete demographic data, major clinical characteristics, and laboratory indicators.

The exclusion criteria were as follows: (1) a diagnosis of heart failure before ASCVD onset; (2) missing key time variables, including the date of ASCVD onset, date of heart failure occurrence, or date of death, or obvious logical inconsistencies in these variables; (3) missing data on the main exposure variable, TC; (4) severe missingness in major covariates that precluded inclusion in multivariable model analyses; (5) incident heart failure occurring before or on the date of baseline TC measurement.

According to the above criteria, a total of 11,479 patients with ASCVD were finally included in the present analysis.

### 2.3 Data collection

The collected data mainly included demographic characteristics, lifestyle factors, medical history, medication history, physical examination parameters, laboratory indicators, and follow-up outcome information.

Demographic characteristics included age and sex. Lifestyle information mainly included smoking status. Medical history included hypertension, diabetes mellitus, dyslipidemia, and ASCVD subtype. Medication information included the use of antihypertensive agents, glucose-lowering agents, lipid-lowering agents, and statins. Information on specific non-statin lipid-lowering therapies, including ezetimibe and PCSK9 inhibitors, was not consistently recorded and therefore could not be analyzed separately. Physical examination parameters included height, weight, body mass index (BMI), and systolic blood pressure (SBP). Laboratory indicators included TC, high-density lipoprotein cholesterol (HDL-C), low-density lipoprotein cholesterol (LDL-C), triglyceride (TG), C-reactive protein (CRP), alanine aminotransferase (ALT), hemoglobin (HGB), serum creatinine, and estimated glomerular filtration rate (eGFR). For laboratory variables used in the baseline and sensitivity analyses, measurements obtained during hospitalization for the index ASCVD event were used preferentially; when unavailable, values from the first and subsequently the second health examination after ASCVD onset were used in sequence.

BMI was calculated as weight divided by height squared and expressed in kg/m^2^. eGFR was calculated based on serum creatinine levels. Hypertension, diabetes mellitus, and dyslipidemia were determined comprehensively according to previous medical history, health examination records, laboratory test results, and relevant medication records.

### 2.4 Exposure variable and grouping

The primary exposure was serum TC measured after ASCVD onset. TC values were selected according to a prespecified hierarchical procedure. Measurements obtained during hospitalization for the index ASCVD event were used preferentially. If an in-hospital TC measurement was unavailable, the TC value obtained at the first health examination after ASCVD onset was used; if this value was also unavailable, the TC value obtained at the second post-ASCVD health examination was used. Only measurements obtained before the occurrence of incident HF were eligible. The selected TC measurement was defined as the baseline TC value, and the date of this measurement was defined as the baseline date. If hospitalization TC was unavailable, the TC value obtained at the closest follow-up examination after ASCVD diagnosis and before HF occurrence was used. The interval between ASCVD diagnosis and post-ASCVD TC measurement was calculated to describe the timing of exposure assessment and was reported in the Results section as the mean interval, consistent with the reporting approach used in previous Kailuan Study analyses. Participants were categorized into five clinically defined TC groups (C1–C5) rather than data-derived quintiles. The thresholds of 5.2 and 6.2 mmol/L correspond to commonly used clinical cut-offs for desirable, borderline-high, and high TC concentrations. Because the present study specifically aimed to evaluate whether both low and high TC levels were associated with incident HF, the lower TC range (<5.2 mmol/L) was further subdivided into three clinically meaningful categories using thresholds of 3.1 and 4.1 mmol/L to distinguish very low, low, and intermediate-low TC levels. The C3 group (4.1–<5.2 mmol/L), representing intermediate TC levels within the clinically desirable range, was selected as the reference category because it corresponded to the lowest observed HF incidence and approximated the nadir of the restricted cubic spline curve, thereby facilitating simultaneous comparison of HF risk associated with both lower and higher TC levels.

### 2.5 Outcome definition and follow-up

The primary outcome of this study was incident heart failure. Incident heart failure was defined as a newly diagnosed heart failure event occurring after the first ASCVD event. Heart failure outcomes were determined comprehensively based on follow-up data, clinical diagnosis and treatment records, hospitalization records, and relevant diagnostic information. HF diagnoses were identified from hospital medical records and confirmed by cardiovascular specialists. Diagnosis was based on clinical symptoms and signs of HF, discharge diagnosis, NYHA or Killip classification, echocardiographic evidence of reduced cardiac function, and/or elevated NT-proBNP levels when available.

The date of baseline TC measurement, rather than the ASCVD event onset date, was used as the start point of follow-up. For participants who developed incident heart failure after baseline, the date of heart failure diagnosis was used as the endpoint date. If any participant died from follow-up period without developing heart failure, such an event was considered as censoring in Cox model analysis and as a competing event in competing risk analysis. In the case where participants did not experience heart failure or death during the follow-up period, the follow-up process would continue until the date when participants were lost to follow-up or up to the end of the study.

Follow-up time was calculated in person-years. The incidence density of incident heart failure was calculated as the number of heart failure events in each group divided by the total person-years of follow-up and was expressed as events per 1,000 person-years.

### 2.6 Covariate definitions

Based on previous studies, clinical relevance, and data availability in this analysis, factors that may influence TC levels and HF risk were selected as covariates. The main covariates included age, sex, smoking status, hypertension, diabetes mellitus, BMI, CRP, eGFR, HDL-C, ALT, HGB, and medication use.

Medication use included the use of lipid-lowering, antihypertensive, and glucose-lowering agents. Considering that lipid levels and lipid-lowering treatment may change during follow-up among patients with ASCVD, TC-related variables during follow-up were further included in additional models to reduce the potential influence of subsequent TC changes on the estimated association between baseline TC levels and HF risk.

### 2.7 Statistical analysis

Statistical software was used for data management and analysis. Continuous variables with an approximately normal distribution were summarized as mean ± standard deviation and compared among groups using analysis of variance. Skewed continuous variables were presented as medians with interquartile ranges, and group differences were assessed using the Kruskal–Wallis rank-sum test. Categorical variables were reported as frequencies and percentages and compared using the χ^2^ test or Fisher’s exact test when appropriate. All statistical tests were two-sided, with *p* < 0.05 indicating statistical significance.

Baseline characteristics were first summarized and compared according to TC categories. For each group, incident heart failure cases, accumulated person-years, and incidence density were calculated. Cox proportional hazards regression was subsequently performed to evaluate the relationship between TC levels and the risk of new-onset heart failure, and the results were reported as *HRs* with corresponding *95%CIs*. Using the C3 group as the reference group, three adjusted models were constructed. Model 1 included sex, hypertension, diabetes mellitus, smoking status, BMI, CRP, age, eGFR, HDL-C, ALT, and HGB as covariates. Model 2 additionally incorporated the use of lipid-lowering, antihypertensive, and glucose-lowering medications. Model 3 additionally included follow-up TC-related variables based on Model 2. Kaplan–Meier curves were generated to visually compare the cumulative incidence of incident heart failure across TC groups, and differences among groups were assessed using the log-rank test.

To assess the robustness of the primary findings, several sensitivity analyses were performed. First, LDL-C and non-HDL-C were examined as alternative lipid exposures using the same Cox proportional hazards models and covariate adjustment strategy as the primary analysis. LDL-C and HDL-C values were selected using the same hierarchical procedure as that used for TC: measurements obtained during hospitalization for the index ASCVD event were used preferentially, followed by measurements obtained at the first and, if necessary, the second health examination after ASCVD onset. Non-HDL-C was calculated as TC minus HDL-C using measurements obtained from the same clinical encounter. Second, to reduce the potential influence of reverse causation from subclinical or rapidly developing HF, the analysis was repeated after excluding participants who developed incident HF within the first year after baseline. Third, the analysis was repeated after excluding participants with baseline malignancy. Both sensitivity analyses used C3 as the reference category and applied the same covariate adjustment strategy as Model 3 of the primary analysis.

Restricted cubic spline models were fitted to examine the nonlinear dose–response association between TC as a continuous variable and incident heart failure. Four knots were placed at the 5th, 35th, 65th, and 95th percentiles of the TC distribution. The reference value was set at 5.0 mmol/L, which was close to the lowest-risk point observed in the spline curve and within the intermediate TC category. The overall association and nonlinearity were tested using Wald tests. The proportional hazards assumption of the Cox regression models was evaluated using Schoenfeld residuals. Both global and variable-specific tests were performed, and a two-sided *p value* >0.05 was considered to indicate no evidence of violation of the proportional hazards assumption. Additional stratified analyses were performed according to baseline statin use and ASCVD subtype. ASCVD subtypes included myocardial infarction, cerebral infarction, and coronary revascularization after non-acute myocardial infarction. Within each ASCVD subtype, Cox proportional hazards models were fitted using C3 as the reference category and applying the same covariate adjustment strategy as Model 3. Subgroup-specific *HR*s with corresponding *95% CI*s were reported. Because these subtype-specific analyses were exploratory and the subgroup sample sizes and event numbers were uneven, no formal interaction test was performed, and the results were not interpreted as definitive evidence of effect modification. All statistical analyses and reporting were conducted in accordance with commonly accepted epidemiological reporting principles to ensure consistency, transparency, and reproducibility.

## 3 Results

### 3.1 Baseline characteristics of patients according to TC levels

A total of 11,479 patients with ASCVD were included in this study and categorized into C1–C5 groups according to baseline TC levels, with 660, 2,750, 4,507, 2,458, and 1,104 patients in each group, respectively. The overall TC level was 4.7 ± 1.2 mmol/L. The mean time interval between ASCVD diagnosis and TC measurement post-ASCVD was 0.60 years. TC levels increased progressively across the groups, with mean values of 2.7 ± 0.4, 3.7 ± 0.3, 4.6 ± 0.3, 5.6 ± 0.3, and 7.0 ± 1.1 mmol/L, respectively, and the difference among groups was statistically significant (*p* < 0.001).

Significant differences were observed across TC groups in age, sex, smoking status, ASCVD subtype, comorbidities, blood pressure, lipid profile, inflammatory markers, hemoglobin levels, and medication use. With increasing TC levels, age showed a decreasing trend, whereas the proportion of women, systolic blood pressure, HDL-C, LDL-C, TG, and HGB levels generally increased. Patients in the low-TC group were relatively older and had higher CRP levels, as well as higher proportions of antihypertensive, lipid-lowering, and statin use. No significant difference in eGFR was observed among the groups (*p* = 0.497).

As shown in Table 1, baseline characteristics differed across TC categories, suggesting that potential confounders, including demographic characteristics, comorbidities, laboratory indicators, and medication use, should be further adjusted for when analyzing the association between TC levels and HF risk.

**Table 1:** Baseline characteristics of patients with ASCVD according to total cholesterol levels.

Variable	Overall *N* = 11,479	C1 N = 660	C2 *N* = 2,750	C3 *N* = 4,507	C4 *N* = 2,458	C5 = 1,104	*p*-value	Max SMD vs C3
TC, mmol/L	4.7 ± 1.2	2.7 ± 0.4	3.7 ± 0.3	4.6 ± 0.3	5.6 ± 0.3	7.0 ± 1.1	<0.001	5.374
Age, years	64.4 ± 9.7	65.6 ± 9.6	65.0 ± 9.9	64.6 ± 9.8	64.1 ± 9.2	62.8 ± 9.0	<0.001	0.191
Sex							<0.001	0.342
Male	10,233 (89.1)	620 (93.9)	2,550 (92.7)	4,083 (90.6)	2,115 (86.0)	865 (78.4)		
Female	1,246 (10.9)	40 (6.1)	200 (7.3)	424 (9.4)	343 (14.0)	239 (21.6)		
Smoking status							<0.001	0.148
Never smoker	5,906 (51.5)	358 (54.2)	1,433 (52.1)	2,229 (49.5)	1,288 (52.4)	598 (54.2)		
Former smoker	925 (8.1)	69 (10.5)	260 (9.5)	363 (8.1)	174 (7.1)	59 (5.3)		
Current smoker	4,648 (40.5)	233 (35.3)	1,057 (38.4)	1915 (42.5)	996 (40.5)	447 (40.5)		
ASCVD subtype							<0.001	0.507
Myocardial infarction	1723 (15.0)	166 (25.2)	470 (17.1)	591 (13.1)	311 (12.7)	185 (16.8)		
Cerebral infarction	8,400 (73.2)	353 (53.5)	1783 (64.8)	3,468 (76.9)	1962 (79.8)	834 (75.5)		
Coronary revascularization after non-acute myocardial infarction	1,356 (11.8)	141 (21.4)	497 (18.1)	448 (9.9)	185 (7.5)	85 (7.7)		
Hypertension	8,794 (76.6)	497 (75.3)	2063 (75.0)	3,439 (76.3)	1912 (77.8)	883 (80.0)	0.009	0.090
Diabetes mellitus	4,461 (38.9)	270 (40.9)	1,000 (36.4)	1,637 (36.3)	1,007 (41.0)	547 (49.5)	<0.001	0.269
Dyslipidemia	6,167 (53.7)	399 (60.5)	1,412 (51.3)	2040 (45.3)	1,438 (58.5)	878 (79.5)	<0.001	0.755
BMI, kg/m^2^	25.6 ± 3.2	25.7 ± 2.9	25.6 ± 3.2	25.6 ± 3.2	25.7 ± 3.2	25.9 ± 3.3	0.015	0.092
SBP, mmHg	144.5 ± 22.1	139.6 ± 20.7	142.3 ± 21.4	144.5 ± 22.0	146.4 ± 22.3	148.6 ± 23.0	<0.001	0.229
HDL-C, mmol/L	1.3 ± 0.4	1.0 ± 0.3	1.2 ± 0.3	1.3 ± 0.4	1.3 ± 0.4	1.5 ± 0.5	<0.001	0.849
LDL-C, mmol/L	2.7 ± 1.2	1.5 ± 0.5	2.0 ± 0.5	2.6 ± 0.6	3.2 ± 0.7	4.0 ± 2.9	<0.001	1.992
TG, mmol/L	1.7 ± 1.4	1.3 ± 0.9	1.3 ± 0.9	1.6 ± 1.2	2.0 ± 1.5	2.7 ± 2.5	<0.001	0.561
eGFR	88.7 ± 21.1	88.3 ± 19.3	88.3 ± 22.2	88.7 ± 21.0	89.3 ± 20.4	88.8 ± 21.1	0.497	0.029
CRP	7.9 ± 15.0	10.4 ± 20.6	7.9 ± 15.6	7.9 ± 14.9	7.5 ± 13.6	7.6 ± 12.2	<0.001	0.139
ALT, U/L	29.0 ± 22.0	30.5 ± 26.8	29.6 ± 23.1	28.4 ± 21.7	28.7 ± 20.9	29.4 ± 19.6	0.049	0.086
HGB, g/L	144.4 ± 17.1	139.1 ± 19.1	142.3 ± 17.9	144.8 ± 16.4	146.4 ± 16.2	147.2 ± 17.1	<0.001	0.320
Antihypertensive medication use	6,605 (57.5)	450 (68.2)	1714 (62.3)	2,493 (55.3)	1,320 (53.7)	628 (56.9)	<0.001	0.268
Lipid-lowering medication use	4,994 (43.5)	380 (57.6)	1,411 (51.3)	1818 (40.3)	936 (38.1)	449 (40.7)	<0.001	0.351
Glucose-lowering medication use	2,355 (20.5)	175 (26.5)	552 (20.1)	855 (19.0)	495 (20.1)	278 (25.2)	<0.001	0.180
Statin use	4,796 (41.8)	369 (55.9)	1,353 (49.2)	1755 (38.9)	891 (36.2)	428 (38.8)	<0.001	0.346

Continuous variables are presented as mean ± SD, and categorical variables are presented as *n* (%). Max SMD vs C3 was calculated as the maximum absolute standardized mean difference comparing each TC group with the C3 reference group. Values ≥0.10 suggest non-negligible imbalance. For categorical variables with multiple levels, the maximum level-specific SMD is reported in the parent variable row. Abbreviations: ASCVD, atherosclerotic cardiovascular disease; TC, total cholesterol; BMI, body mass index; SBP, systolic blood pressure; HDL-C, high-density lipoprotein cholesterol; LDL-C, low-density lipoprotein cholesterol; TG, triglyceride; eGFR, estimated glomerular filtration rate; CRP, C-reactive protein; ALT, alanine aminotransferase; HGB, hemoglobin.

### 3.2 Incidence of incident heart failure according to TC levels

During follow-up, 610 cases of incident heart failure occurred among 11,479 patients with ASCVD, with an incidence proportion of 5.31%. The total follow-up time was 71,406.7 person-years, and the overall incidence density was 8.54 per 1,000 person-years. After stratification by baseline TC levels, the numbers of heart failure events in the C1–C5 groups were 54, 146, 209, 121, and 80, respectively. The corresponding incidence densities were 14.35, 8.46, 7.32, 7.89, and 12.34 per 1,000 person-years, respectively. As shown in Table 2, the C3 group had the lowest incidence density, whereas both the low-TC group (C1) and the high-TC group (C5) had higher incidence densities than the intermediate TC groups, suggesting a potential nonlinear association between baseline TC levels and HF risk in patients with ASCVD.

**Table 2:** Incident heart failure events and incidence density according to TC levels.

TC category	No. of patients	HF events	Person-years	Incidence rate per 1,000 person-years	95% CI
C1 (<3.1)	660	54	3763.9	14.35	10.78–18.73
C2 (3.1–<4.1)	2,750	146	17,258	8.46	7.14–9.96
C3 (4.1–<5.2)	4,507	209	28,565	7.32	6.36–8.37
C4 (5.2–<6.2)	2,458	121	15,339	7.89	6.55–9.43
C5 (≥6.2)	1,104	80	6480.8	12.34	9.78–15.38

The C3 group, defined as TC 4.1–<5.2 mmol/L, was used as the reference group in the Cox proportional hazards regression models.

Kaplan–Meier analysis demonstrated significant differences in the cumulative incidence of incident heart failure across the five TC categories (log-rank *p* < 0.0001; Supplementary Figure 1). The C3 group exhibited the lowest cumulative incidence throughout follow-up, whereas both the C1 and C5 groups showed consistently higher cumulative incidence than the intermediate TC groups, supporting the U-shaped association observed in the multivariable Cox regression and restricted cubic spline analyses.

### 3.3 Cox regression analysis of the association between TC levels and the HF risk

Because the primary objective of this study was to evaluate whether both low and high TC levels were associated with excess HF risk, C3, which represented the clinically desirable TC range and showed the lowest observed HF incidence, was selected as the reference category.

Compared with the C3 group, participants in the C1 and C5 groups had significantly higher HF risk across all three Cox models, whereas no significant associations were observed for the C2 or C4 groups. In the fully adjusted model (Model 3), the *HRs* were 1.578 (*95% CI* 1.160–2.148; *p* = 0.004) for C1 and 1.509 (*95% CI* 1.150–1.979; *p* = 0.003) for C5. In contrast, C2 (*HR* 1.043, *95% CI* 0.841–1.294; *p* = 0.700) and C4 (*HR* 1.068, *95% CI* 0.851–1.341; *p* = 0.570) were not associated with increased HF risk. Similar findings were observed in Models 1 and 2 (Table 3).

**Table 3:** Cox regression analysis of the association between TC levels and HF risk.

TC category	Events/total	Model 1 HR (95% CI)	*p*-value	Model 2 HR (95% CI)	*p*-value	Model 3 HR (95% CI)	*p*-value
C1 (<3.1)	54/660	1.802 (1.331–2.440)	<0.001	1.512 (1.115–2.052)	0.008	1.578 (1.160–2.148)	0.004
C2 (3.1–<4.1)	146/2750	1.119 (0.904–1.385)	0.301	1.019 (0.822–1.262)	0.867	1.043 (0.841–1.294)	0.7
C3 (4.1–<5.2)	209/4507	Reference	—	Reference	—	Reference	—
C4 (5.2–<6.2)	121/2458	1.059 (0.845–1.327)	0.617	1.095 (0.873–1.372)	0.433	1.068 (0.851–1.341)	0.57
C5 (≥6.2)	80/1104	1.594 (1.223–2.078)	<0.001	1.596 (1.225–2.080)	<0.001	1.509 (1.150–1.979)	0.003

The proportional hazards assumption was satisfied for the final Cox model. Schoenfeld residual tests showed no significant violation for either the global model or any individual covariate (all *p* > 0.05).

### 3.4 Nonlinear association between TC levels and HF risk

**Figure 1:**
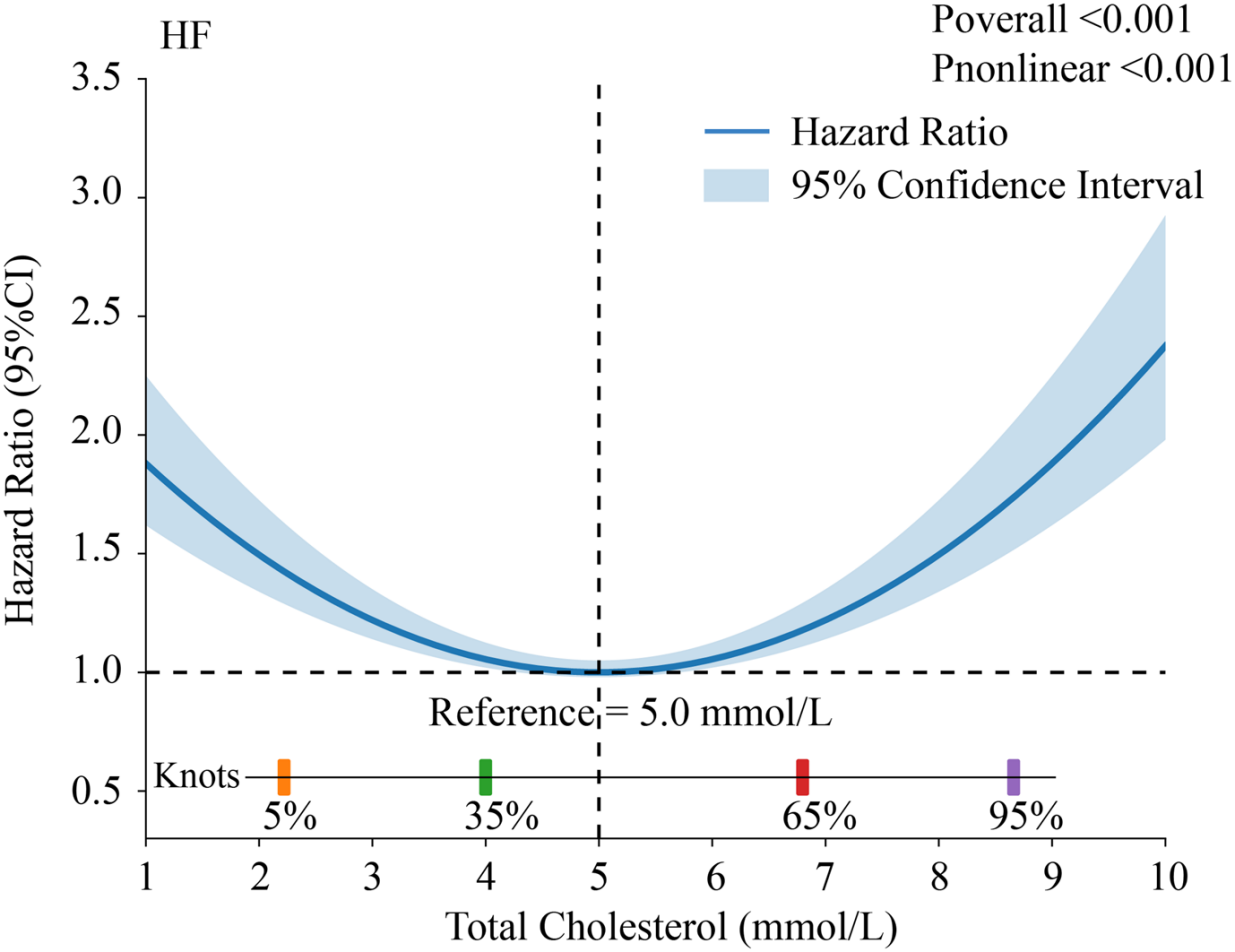
Restricted cubic spline analysis of the association between baseline TC levels and incident heart failure.

To further evaluate the dose–response relationship between baseline TC levels and HF risk, TC was included as a continuous variable in restricted cubic spline models. The results showed significant overall and nonlinear associations between TC levels and HF risk (*P* for overall association <0.001; *P* for nonlinearity <0.001). The restricted cubic spline curve showed an approximately U-shaped pattern. The risk of heart failure gradually decreased as TC levels increased from low to intermediate levels and reached a relatively low level at approximately 5.0 mmol/L. However, the risk increased again with further elevation of TC levels. These findings suggest that both lower and higher TC levels may be associated with a higher risk of incident heart failure in patients with ASCVD, and this trend was generally consistent with the grouped analysis and Cox regression results (Figure 1).

Adjusted hazard ratios and 95% confidence intervals were derived from the restricted cubic spline model. Four knots were placed at the 5th, 35th, 65th, and 95th percentiles of the TC distribution. The reference value was 5.0 mmol/L. The overall association and nonlinearity were statistically significant (*Poverall* <0.001; *Pnonlinearity* <0.001).

### 3.5 Competing risk model analysis

Considering that death may preclude the observation of incident heart failure, Fine–Gray subdistribution hazard models were performed with death treated as the competing event to further evaluate the association between TC levels and incident heart failure. Using the C3 group as the reference, the results showed that the risks of incident heart failure were significantly higher in both the low-TC group (C1) and the high-TC group (C5) than in the C3 group across different adjusted models, whereas no statistically significant differences were observed in the C2 and C4 groups.

In Model 1, the *SHRs* for the C1 and C5 groups were 1.867 (95% *CI*: 1.375–2.535, *p* < 0.001) and 1.541 (95% *CI*: 1.182–2.010, *p* = 0.001), respectively. After further adjustment for medication use, the risks remained significantly elevated in the C1 and C5 groups, with *SHR* of 1.550 (95% *CI*: 1.137–2.115, *p* = 0.006) and 1.546 (95% *CI*: 1.186–2.017, *p* = 0.001), respectively. After further inclusion of follow-up TC-related variables, the risks in the C1 and C5 groups remained higher than those in the C3 group, with *SHR* of 1.618 (95% *CI*: 1.180–2.218, *p* = 0.003) and 1.468 (95% *CI*: 1.116–1.930, *p* = 0.006), respectively.

Overall, the results of the Fine–Gray competing-risk models were generally consistent with those of the primary Cox regression analysis, supporting the robustness of the observed associations (Table 4).

**Table 4:** Fine–Gray subdistribution hazard model for the association between TC levels and incident heart failure.

TC category	Model 1 SHR (95% CI)	*p*-value	Model 2 SHR (95% CI)	*p*-value	Model 3 SHR (95% CI)	*p*-value
C1 (<3.1)	1.867 (1.375–2.535)	<0.001	1.550 (1.137–2.115)	0.006	1.618 (1.180–2.218)	0.003
C2 (3.1–<4.1)	1.148 (0.927–1.421)	0.206	1.040 (0.839–1.289)	0.72	1.066 (0.859–1.322)	0.564
C3 (4.1–<5.2)	Reference	—	Reference	—	Reference	—
C4 (5.2–<6.2)	1.063 (0.849–1.332)	0.593	1.099 (0.877–1.378)	0.411	1.074 (0.854–1.350)	0.541
C5 (≥6.2)	1.541 (1.182–2.010)	0.001	1.546 (1.186–2.017)	0.001	1.468 (1.116–1.930)	0.006

The C3 group, defined as TC 4.1–<5.2 mmol/L, was used as the reference group. Model 1 was adjusted for sex, hypertension, diabetes mellitus, smoking status, BMI, CRP, age, eGFR, HDL-C, ALT, and hemoglobin. Model 2 was further adjusted for the use of lipid-lowering, antihypertensive, and glucose-lowering medications based on Model 1. Model 3 additionally included follow-up TC-related variables based on Model 2. TC, total cholesterol; BMI, body mass index; CRP, C-reactive protein; eGFR, estimated glomerular filtration rate; HDL-C, high-density lipoprotein cholesterol; HGB, hemoglobin; ALT, alanine aminotransferase; SHR, subdistribution hazard ratio; CI, confidence interval.

### 3.6 Sensitivity analyses using alternative lipid parameters

To assess the robustness of the primary findings, additional Cox regression analyses were performed using LDL-C, non-HDL-C, standardized TC, and continuous TC as alternative lipid exposures. Compared with the corresponding reference categories, both the lowest and highest categories of LDL-C and non-HDL-C were significantly associated with increased HF risk after multivariable adjustment. Standardized TC was not significantly associated with HF risk per 1-SD increase (*HR* = 1.012, *95% CI*: 0.931–1.100; *p* = 0.778), whereas continuous TC showed a weak positive association with HF risk per 1 mmol/L increase (*HR* = 1.067, *95% CI*: 1.001–1.138; *p* = 0.048). These findings should be interpreted together with the significant nonlinear association observed in the restricted cubic spline analysis, suggesting that the association between TC and HF risk may not be adequately characterized by a simple linear model (Supplementary Tables S1, S2).

### 3.7 Stratified analysis according to statin use

In participants receiving baseline statin therapy, compared with the C3 group, only the C5 group remained significantly associated with an increased risk of incident HF (adjusted *HR* = 1.449, *95% CI:* 1.018–2.063, *p* = 0.040), whereas no significant associations were observed for C1, C2, or C4.

Among participants not receiving statins at baseline, both the C1 and C5 groups showed significantly higher risks of incident HF compared with the C3 group, with adjusted *HRs* of 2.396 (*95% CI:* 1.440–3.987, *p* = 0.0008) and 1.569 (*95% CI:* 1.019–2.416, *p* = 0.0408), respectively. No significant associations were observed for C2 or C4.

Overall, although the association between very low TC and HF risk appeared stronger among non-statin users, no statistically significant interaction between TC categories and baseline statin use was detected (*P* for interaction > 0.05).

### 3.8 Additional sensitivity analyses

After excluding participants who developed incident HF within the first year after baseline, the associations of both very low and high TC levels with subsequent HF remained statistically significant. Compared with the C3 group, the adjusted *HR* was 1.802 (*95% CI*: 1.178–2.756; *p* = 0.0066) for C1 and 1.936 (*95% CI*: 1.338–2.802; *p* = 0.0005) for C5. No statistically significant associations were observed for C2 or C4, with adjusted *HR*s of 1.267 (*95% CI*: 0.949–1.693; *p* = 0.1091) and 1.130 (*95% CI*: 0.823–1.553; *p* = 0.4492), respectively.

After excluding participants with baseline malignancy, the results also remained consistent with the primary analysis. Compared with C3, the adjusted *HR*s were 1.538 (*95% CI*: 1.118–2.117; *p* = 0.0082) for C1 and 1.560 (*95% CI*: 1.179–2.063; *p* = 0.0018) for C5. The associations for C2 and C4 were not statistically significant, with adjusted *HR*s of 1.062 (*95% CI*: 0.850–1.325; *p* = 0.5972) and 1.098 (*95% CI*: 0.868–1.388; *p* = 0.4363), respectively. Detailed results are presented in Supplementary Table S5.

### 3.9 Stratified analysis according to ASCVD subtype

The association between TC categories and incident HF was further examined according to ASCVD subtype. Among patients with myocardial infarction, both C1 and C5 were associated with significantly higher HF risk than C3, with adjusted *HR*s of 1.997 (*95% CI*: 1.285–3.104; *p* = 0.0021) and 1.748 (*95% CI*: 1.128–2.709; *p* = 0.0125), respectively. The associations for C2 and C4 were not statistically significant, with adjusted *HR*s of 1.015 (*95% CI*: 0.696–1.481; *p* = 0.9375) and 1.347 (*95% CI*: 0.920–1.973; *p* = 0.1256), respectively.

Among patients with cerebral infarction, the adjusted *HR*s were 1.477 (*95% CI*: 0.875–2.492; *p* = 0.1443) for C1 and 1.431 (*95% CI*: 0.971–2.110; *p* = 0.0702) for C5. Although both point estimates were greater than 1, neither association reached statistical significance. No statistically significant associations were observed for C2 or C4, with adjusted *HR*s of 1.157 (*95% CI*: 0.850–1.576; *p* = 0.3548) and 1.009 (*95% CI*: 0.736–1.383; *p* = 0.9553), respectively.

Among patients who underwent coronary revascularization after non-acute myocardial infarction, none of the TC categories was significantly associated with incident HF compared with C3. The adjusted *HR*s were 0.467 (*95% CI*: 0.177–1.231; *p* = 0.1235) for C1, 0.765 (*95% CI*: 0.458–1.279; *p* = 0.3073) for C2, 0.736 (*95% CI*: 0.365–1.486; *p* = 0.3928) for C4, and 1.121 (*95% CI*: 0.479–2.625; *p* = 0.7921) for C5.

These subtype-specific findings should be interpreted cautiously because the analyses were exploratory and some estimates had wide confidence intervals, particularly in the coronary revascularization subgroup. No formal interaction test was performed; therefore, the results should not be interpreted as demonstrating statistically significant differences in the TC–HF association across ASCVD subtypes. Detailed results are presented in Supplementary Table S6.

## 4 Discussion

In this cohort of patients with ASCVD, baseline TC levels showed an approximately U-shaped association with incident heart failure. Compared with the intermediate TC group, both the very low-TC and high-TC groups had higher HF risk after multivariable adjustment. This pattern was supported by restricted cubic spline and competing-risk analyses and remained robust after excluding HF events occurring within the first year after baseline and after excluding participants with baseline malignancy. These findings suggest that TC may serve as an epidemiological marker of HF risk in patients with ASCVD, but the association should not be interpreted as evidence that lowering TC directly increases HF risk. Therefore, the intermediate TC category (4.1–<5.2 mmol/L) served as the clinical reference because it represented the lowest-risk range rather than the lowest cholesterol concentration.

Previous studies on the relationship between lipid levels and the risk of heart failure have yielded inconsistent findings. In this analysis, a nonlinear association was observed between TC levels and incident heart failure, which is generally consistent with recent evidence suggesting that the relationship between lipid levels and heart failure risk is not simply linear (18, 19). Lee et al., using data from the Korean National Health Information Database, found that the association between LDL-C and the risk of heart failure may be influenced by diabetes duration. Similar patterns were also observed for TC and non-HDL-C, suggesting that, in the context of metabolic abnormalities or chronic disease progression, the relationship between lipid levels and heart failure risk may be jointly affected by underlying disease status, nutritional condition, and treatment factors (20). Dawson et al. reported that individuals with established ASCVD require specific risk prediction models for heart failure, indicating that patients with ASCVD represent a population at high risk of heart failure (21). In addition, Huang et al. found that remnant cholesterol was positively associated with the risk of heart failure, suggesting that some atherogenic lipid components may contribute to the development of heart failure (22). Compared with previous studies, this analysis focused specifically on patients with ASCVD and used TC as an epidemiological exposure indicator to evaluate its association with incident heart failure. It should be emphasized that TC is not intended to replace LDL-C, non-HDL-C, ApoB, or remnant cholesterol as therapeutic targets in ASCVD management. Rather, TC was used because it is routinely available across repeated health examinations and reflects the combined cholesterol content of several lipoprotein fractions. The additional analyses using LDL-C and non-HDL-C helped evaluate whether the observed association was consistent across related lipid parameters. These findings suggest that both high and low TC levels may serve as markers of increased heart failure risk in patients with ASCVD. In exploratory analyses stratified by ASCVD subtype, the associations of both C1 and C5 with incident HF were statistically significant among patients with myocardial infarction. Among patients with cerebral infarction, the point estimates for C1 and C5 were also greater than 1, although the corresponding associations did not reach statistical significance. No statistically significant associations were observed in the coronary revascularization subgroup, in which several estimates had wide confidence intervals. These patterns may partly reflect differences in underlying myocardial injury, subgroup sample sizes, and event numbers. Because no formal interaction test was performed, these findings should not be interpreted as definitive evidence that the association between TC and HF differs across ASCVD subtypes.

The increased HF risk observed among patients with high TC levels is biologically plausible. Elevated TC usually reflects increased concentrations of LDL-containing lipoproteins and remnant lipoproteins, which promote endothelial dysfunction, oxidative stress, vascular inflammation, and accelerated atherosclerotic plaque progression (23–25). Progressive coronary atherosclerosis may lead to recurrent myocardial ischemia, myocardial infarction, adverse ventricular remodeling, and eventually heart failure (26, 27). Beyond vascular injury, excessive cholesterol accumulation may also contribute to myocardial lipotoxicity (28, 29). Experimental studies have shown that abnormal lipid deposition within cardiomyocytes can impair mitochondrial function, disturb myocardial energy metabolism, increase reactive oxygen species production, and activate inflammatory and fibrotic signaling pathways, ultimately promoting myocardial dysfunction and heart failure progression (30–32). In addition, hypercholesterolemia frequently coexists with hypertension, diabetes, obesity, and insulin resistance, which further aggravate myocardial fibrosis, microvascular dysfunction, and chronic low-grade inflammation (33–35).

By contrast, the mechanisms underlying the association between low TC and HF are likely to be different. Low TC should not be interpreted as a direct cause of heart failure. Instead, it may serve as a marker of poorer overall health status, advanced disease burden, or altered metabolic homeostasis. Previous studies have described the so-called “cholesterol paradox,” in which lower cholesterol concentrations are associated with worse outcomes in patients with chronic heart failure or other severe chronic illnesses (36, 37). Several hypotheses have been proposed, including chronic inflammation, cachexia, impaired hepatic cholesterol synthesis, increased catabolism, and neurohormonal activation (38–40). Persistent inflammation may suppress cholesterol production while simultaneously accelerating myocardial remodeling and ventricular dysfunction (41–43). Furthermore, metabolic depletion and impaired myocardial energy utilization in advanced cardiovascular disease may also contribute to lower circulating cholesterol concentrations (44, 45). However, because nutritional markers such as albumin and prealbumin were unavailable in this analysis, these mechanisms should be regarded as biologically plausible hypotheses rather than confirmed explanations.

Several limitations should be acknowledged. First, this was an observational study. Although multivariable Cox regression, restricted cubic spline analysis, and competing risk models were used for statistical adjustment, the influence of residual confounding could not be completely excluded, and causal inference cannot be directly established. Second, TC levels may be influenced by multiple factors, including diet, medication adherence, lipid-lowering treatment intensity, liver function, nutritional status, and systemic inflammation. Although baseline lipid-lowering medication use was included in the multivariable models and stratified analyses according to statin use were performed, detailed information on statin intensity, cumulative dose, medication adherence, treatment changes during follow-up, and the separate use of ezetimibe or PCSK9 inhibitors was not consistently available. Therefore, ezetimibe and PCSK9 inhibitors could not be evaluated separately, and residual confounding related to the type, intensity, duration, and changes in lipid-lowering therapy remains possible. In addition, several established predictors of heart failure, including left ventricular ejection fraction (LVEF), BNP/NT-proBNP, infarct size, Killip class, coronary revascularization procedures (PCI/CABG), and guideline-directed cardiovascular therapies such as ACEI/ARB/ARNI, *β*-blockers, SGLT2 inhibitors, and mineralocorticoid receptor antagonists, were not consistently available in the present database and therefore could not be included in the adjustment models. Because these factors may influence both lipid management and the subsequent risk of heart failure, residual confounding could not be fully eliminated. Third, reverse causality may partly explain the association observed in the low-TC group. Some patients may have developed lower TC levels after ASCVD onset because of chronic disease burden, malnutrition, inflammation-related wasting, or prior intensive lipid-lowering treatment. Therefore, the association between low TC levels and increased heart failure risk should be interpreted with caution. Although the findings remained robust after excluding HF events occurring within the first year and after excluding participants with baseline malignancy, other severe systemic conditions were not consistently captured in the database and could not be reliably incorporated into a broader exclusion analysis. Consequently, reverse causation and confounding by unmeasured severe illness cannot be completely excluded. Fourth, this study mainly used baseline TC levels as the primary exposure variable. Although follow-up TC-related variables were further included for adjustment, long-term lipid trajectories, achievement of lipid-lowering targets, and lipid variability could not be fully captured. In addition, although LDL-C and non-HDL-C were examined in sensitivity analyses, ApoB and directly measured remnant cholesterol were unavailable in the present dataset. Therefore, we could not fully compare TC with all guideline-recommended lipid targets. Moreover, direct nutritional indicators, such as albumin, prealbumin, and weight loss, were not available. Thus, explanations related to malnutrition, frailty, or depleted health status should be interpreted as biologically plausible hypotheses rather than confirmed mechanisms. Future studies incorporating serial lipid measurements, ApoB, remnant cholesterol, nutritional markers, and comprehensive heart failure–related clinical variables are warranted to further validate our findings. Finally, the ASCVD subtype analyses were exploratory, and the subgroup sample sizes and event numbers were uneven. Some estimates had wide confidence intervals, particularly among patients who underwent coronary revascularization after non-acute myocardial infarction. A formal interaction test was not performed, and the observed subtype-specific patterns should therefore not be interpreted as definitive evidence of effect modification.

From a clinical perspective, the present findings should not be interpreted as evidence that guideline-recommended LDL-C lowering should be relaxed in patients with ASCVD. LDL-C remains the primary therapeutic target for secondary prevention according to current international guidelines. Although intensive lipid lowering remains essential for secondary prevention of recurrent ASCVD events, HF is a distinct outcome from recurrent atherosclerotic events. Therefore, unexpectedly low cholesterol levels should be interpreted cautiously as a potential marker of poor health status, metabolic depletion, or intensive treatment history rather than as evidence against guideline-directed lipid-lowering therapy. Instead, unexpectedly low TC levels should be interpreted within the broader clinical context. In patients with markedly low TC levels, clinicians should consider comprehensive evaluation of nutritional status, inflammatory burden, frailty, comorbid conditions, and the intensity and adherence of lipid-lowering therapy, rather than attributing low TC solely to successful lipid control. Such an integrated assessment may help identify patients at increased risk of subsequent heart failure and facilitate more individualized management.

In conclusion, this study found a nonlinear association between serum TC levels and HF risk in patients with ASCVD, with both lower and higher TC levels associated with increased heart failure risk. These findings suggest that lipid assessment in patients with ASCVD should not focus only on the atherosclerotic risk represented by high cholesterol levels, but should also consider the potential health risks reflected by low TC levels. In clinical practice, unexpectedly low TC levels should not automatically be regarded as an indicator of optimal lipid control. Instead, they should prompt a comprehensive clinical assessment integrating cardiovascular risk, nutritional status, inflammatory burden, frailty, medication use, and comorbid conditions to better identify patients at increased risk of heart failure while maintaining guideline-directed lipid-lowering therapy.

## Data Availability

The data analyzed in this study is subject to the following licenses/restrictions: the datasets are not publicly available due to privacy and ethical restrictions. Access to the data requires reasonable request to the corresponding author and permission from the Kailuan Study investigators. Requests to access the datasets should be directed to QZ, zhangqi_08260803@163.com.
