# Supplementary material for: Association between serum total cholesterol levels and the risk of incident heart failure in patients with atherosclerotic cardiovascular disease
Source: Front Med (Lausanne). 2026 Aug 28;13:1884333. doi: 10.3389/fmed.2026.1884333 (PMC13561770; doi:10.3389/fmed.2026.1884333)
Supplement: Supplementary file 1 [file Data_Sheet_1.docx]

**Supplementary Table S1. Sensitivity analyses using alternative lipid parameters**

| **Lipid parameter** | **Category** | **Adjusted *HR (95% CI)*** | ***P value*** |
| --- | --- | --- | --- |
| LDL-C | LDL1 | 1.762 (1.301–2.387) | <0.001 |
| LDL-C | LDL2 | 1.115 (0.900–1.380) | 0.319 |
| LDL-C | LDL3 | Reference | — |
| LDL-C | LDL4 | 1.063 (0.848–1.332) | 0.597 |
| LDL-C | LDL5 | 1.598 (1.226–2.084) | <0.001 |
| Non-HDL-C | Non-HDL1 | 1.476 (1.088–2.002) | 0.013 |
| Non-HDL-C | Non-HDL2 | 1.014 (0.819–1.257) | 0.897 |
| Non-HDL-C | Non-HDL3 | Reference | — |
| Non-HDL-C | Non-HDL4 | 1.099 (0.877–1.378) | 0.413 |
| Non-HDL-C | Non-HDL5 | 1.602 (1.229–2.088) | <0.001 |

**Note:** Hazard ratios were estimated using Cox proportional hazards models with the same covariate adjustment strategy as the primary analysis. LDL-C, low-density lipoprotein cholesterol; non-HDL-C, non-high-density lipoprotein cholesterol; *HR*, hazard ratio; *CI*, confidence interval.

**Supplementary Table S2. Sensitivity analyses using continuous and standardized TC**

| **Exposure variable** | **Unit** | **Adjusted *HR (95% CI)*** | ***P value*** |
| --- | --- | --- | --- |
| Standardized TC | Per 1-SD increase | 1.012 (0.931–1.100) | 0.778 |
| Continuous TC | Per 1 mmol/L increase | 1.067 (1.001–1.138) | 0.048 |

**Note:** Hazard ratios were estimated using Cox proportional hazards models. TC, total cholesterol; SD, standard deviation; HR, hazard ratio; CI, confidence interval.

**Supplementary Table S3. Stratified analysis among statin users**

| **TC category** | **Adjusted *HR (95% CI)*** | ***P value*** |
| --- | --- | --- |
| C1 | 1.282 (0.870–1.888) | 0.2089 |
| C2 | 1.033 (0.793–1.346) | 0.8085 |
| C3 | Reference | — |
| C4 | 1.102 (0.822–1.477) | 0.5178 |
| C5 | 1.449 (1.018–2.063) | 0.0395 |

**Supplementary Table S4. Stratified analysis among non-statin users**

| **TC category** | **Adjusted *HR (95% CI)*** | ***P value*** |
| --- | --- | --- |
| C1 | 2.396 (1.440–3.987) | 0.0008 |
| C2 | 1.083 (0.744–1.576) | 0.6788 |
| C3 | Reference | — |
| C4 | 1.030 (0.718–1.478) | 0.8734 |
| C5 | 1.569 (1.019–2.416) | 0.0408 |

**Supplementary Table S5. Sensitivity analyses excluding early heart failure events and baseline malignancy**

| **Sensitivity analysis** | **TC category** | **Adjusted *HR* (*95% CI*)** | ***P* value** |
| --- | --- | --- | --- |
| Excluding incident HF within 1 year after baseline | C1 (<3.1 mmol/L) | 1.802 (1.178–2.756) | 0.0066 |
|  | C2 (3.1–<4.1 mmol/L) | 1.267 (0.949–1.693) | 0.1091 |
|  | C3 (4.1–<5.2 mmol/L) | Reference | — |
|  | C4 (5.2–<6.2 mmol/L) | 1.130 (0.823–1.553) | 0.4492 |
|  | C5 (≥6.2 mmol/L) | 1.936 (1.338–2.802) | 0.0005 |
| Excluding participants with baseline malignancy | C1 (<3.1 mmol/L) | 1.538 (1.118–2.117) | 0.0082 |
|  | C2 (3.1–<4.1 mmol/L) | 1.062 (0.850–1.325) | 0.5972 |
|  | C3 (4.1–<5.2 mmol/L) | Reference | — |
|  | C4 (5.2–<6.2 mmol/L) | 1.098 (0.868–1.388) | 0.4363 |
|  | C5 (≥6.2 mmol/L) | 1.560 (1.179–2.063) | 0.0018 |

**Note:** *HR*s were estimated using Cox proportional hazards models with C3 as the reference category. Models were adjusted for sex, hypertension, diabetes mellitus, smoking status, body mass index, C-reactive protein, age, estimated glomerular filtration rate, high-density lipoprotein cholesterol, alanine aminotransferase, hemoglobin, lipid-lowering medication use, antihypertensive medication use, glucose-lowering medication use, and follow-up TC-related variables. HF, heart failure; TC, total cholesterol; *HR*, hazard ratio; *CI*, confidence interval.

**Supplementary Table S6. Association between TC categories and incident heart failure according to ASCVD subtype**

| **ASCVD subtype** | **TC category** | **Adjusted *HR* (*95% CI*)** | ***P* value** |
| --- | --- | --- | --- |
| Myocardial infarction | C1 (<3.1 mmol/L) | 1.997 (1.285–3.104) | 0.0021 |
|  | C2 (3.1–<4.1 mmol/L) | 1.015 (0.696–1.481) | 0.9375 |
|  | C3 (4.1–<5.2 mmol/L) | Reference | — |
|  | C4 (5.2–<6.2 mmol/L) | 1.347 (0.920–1.973) | 0.1256 |
|  | C5 (≥6.2 mmol/L) | 1.748 (1.128–2.709) | 0.0125 |
| Cerebral infarction | C1 (<3.1 mmol/L) | 1.477 (0.875–2.492) | 0.1443 |
|  | C2 (3.1–<4.1 mmol/L) | 1.157 (0.850–1.576) | 0.3548 |
|  | C3 (4.1–<5.2 mmol/L) | Reference | — |
|  | C4 (5.2–<6.2 mmol/L) | 1.009 (0.736–1.383) | 0.9553 |
|  | C5 (≥6.2 mmol/L) | 1.431 (0.971–2.110) | 0.0702 |
| Coronary revascularization after non-acute myocardial infarction | C1 (<3.1 mmol/L) | 0.467 (0.177–1.231) | 0.1235 |
|  | C2 (3.1–<4.1 mmol/L) | 0.765 (0.458–1.279) | 0.3073 |
|  | C3 (4.1–<5.2 mmol/L) | Reference | — |
|  | C4 (5.2–<6.2 mmol/L) | 0.736 (0.365–1.486) | 0.3928 |
|  | C5 (≥6.2 mmol/L) | 1.121 (0.479–2.625) | 0.7921 |

**Note:** *HR*s were estimated separately within each ASCVD subtype using Cox proportional hazards models with C3 as the reference category. Models were adjusted using the same covariate strategy as Model 3 of the primary analysis. Because the subtype-specific analyses were exploratory and subgroup sample sizes and event numbers were uneven, no formal interaction test was performed. ASCVD, atherosclerotic cardiovascular disease; TC, total cholesterol; HF, heart failure; *HR*, hazard ratio; *CI*, confidence interval.

**Supplementary Figure 1. Kaplan–Meier curves for incident heart failure according to baseline TC categories.**
